# Supplementary material for: Memory justifications provide valid indicators of retrieval accuracy across time
Source: Commun Psychol. 2025 Dec 31;4:10. doi: 10.1038/s44271-025-00378-4 (PMC12820377; doi:10.1038/s44271-025-00378-4)
Supplement: Supplementary file 2 — Supplementary Information [file 44271_2025_378_MOESM2_ESM.pdf]

Supplementary Information

Supplementary Note 1 | Pilot study 1

Method

Participants

The study included 33 students from Ben-Gurion University of the Negev, Beer-Sheva, Israel (26 women), aged 21-33 years (Mean = 23.9). Participants were compensated with either course credit or monetary award. All participants were native Hebrew speakers, with normal or corrected-to-normal vision, and with no diagnosis of attention disorders or other neurological deficits.

Materials

Lists

The experiment was constructed of 24 lists of 16 words each. A fixed list of 16 words served as a practice-list. It was presented before the first block and was not included in the analyses. Words were nouns, verbs and adjectives, 3-9 letters long, selected from a Hebrew pool of free association norms, which contains 800 words [1]. These words were included in a semantic network [2] thus enabling to derive semantic distances between words [3]. The lists were constructed such that temporal and semantic contributions to recall can be dissociated. For each list, 30 different ordering schemes of the words within the list were created. Each participant was randomly assigned to one of these ordering schemes. These lists were used in previous studies [4] and yielded reliable temporal and semantic clustering effects.

Justifications

Participants' self-reports of their recall process, or "justifications", were reported in Hebrew. The procedure was inspired by previous studies in which participants reported introspective reflections from the study phase ("Internal Mentations" [5], "Justifications" [6]). In the current study, following each recall test phase, participants were asked to describe the information they used to recall the word. Justifications were recalled and typed by participants who were cued with each word recalled during the word recall phase. The justifications were then manually coded for analysis into one of two exclusive groups: With-Justification (J+) (i.e., an item recalled with a specific description), or Without-Justification (J-) (i.e., an item recalled without an informative description). Justifications were then also divided into eight categories reflecting the specific recall strategy utilized. The categories are based on classifications in a previous study regarding strategies used in a semantic fluency task [7]. Table 1 presents the eight categories and a brief explanation of each. The eight categories are not mutually exclusive. A justification might relate to more than one category.

Category	Sub-Category	Sub-Category meaning	Justification example	Word relating
Justification -	-	No Justification	I cannot remember any reason for recalling the word	Bag
Justification +	Contiguous	Relating current word to a word learned sequentially	I associated it with the word 'war' which came after it	Exit
	Semantic	Relating current item to a word with semantic similarity (or a shared semantic group, i.e., 'animals')	Relates to bullies* (*"Bully" being another word retrieved from the list)	Fist
	Important to self	Relating current word to participant's life, experiences, etc.	My favorite animal	Dog
	Location in list	Relating current word to a specific location in list (i.e., first, last)	Was the first word on the list	Peach
	Plot	(Explicitly) recalling a narrative or story including the current word	I told a story about a girl called dot	Dot

	Phonological	Relating current word to a similar sounding word (i.e., rhyme or alliteration)	Patch rhymes with catch	Patch
	Rare	Current word stood out as unusual or rare.	Unusual word	Tyke

Justifications' detail

To further explore the effects of time on justifications, each justification was coded manually for its level of detail. The counts of details were based on the Autobiographical Interview [8,9], in which the number of details providing distinct pieces of information are counted. Furthermore, details are classified as internal or external to the event described. Regarding justifications, this classification pertained to details that relate directly to the relevant word recalled, and those that branched out to unrelated topics. Thus, the justifications' details were classified as either word-related or word-unrelated. In addition, nouns in each justification were classified as abstract or concrete, to count the number of abstract/concrete nouns in each justification's text. Levels of language abstraction may be reflective of transformation from a contextually-detailed memory to a verbatim gist memory [10,11], and hence the abstract/concrete classification.

Justifications' linguistic content

To assess the changes in justification over time. The words used in the justifications were assessed using the python *Scattertext* package [12]. Word frequencies were calculated in each delay condition to examine which words differentiate between conditions. Word scores were compared across conditions using cohen's d scores, using the difference in scores by delay condition using the pooled standard error.

Table A1. Justification sub-categories Detailed (Real examples given by study participants; translated from Hebrew).

Experimental procedure

The experiment consisted of a total number of 24 blocks divided into two sessions of 12 blocks each, with 5-7 days between the first and second sessions. Each block consisted of 4 stages: I. study phase, II. Distraction task, III. Free-recall, IV. Self-report of justifications. Figure A1 illustrates the experimental sequence for a single block.

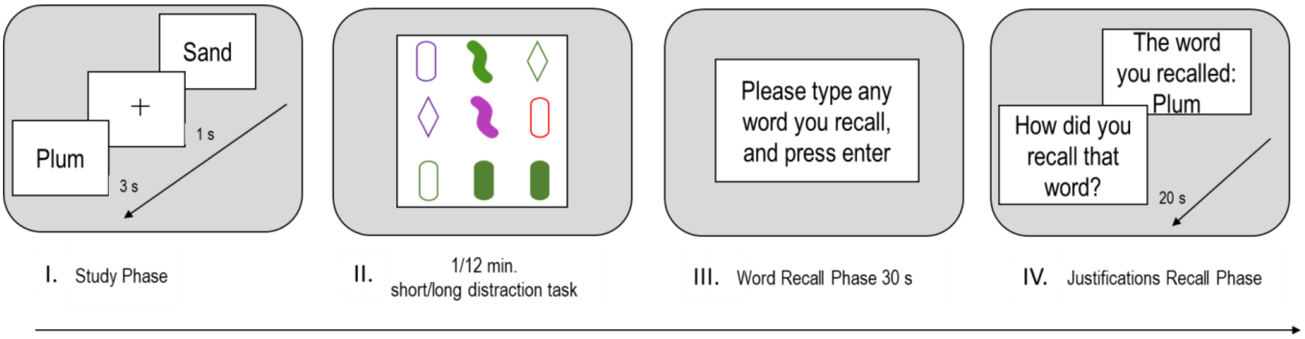

Figure A1. Illustration of the experimental sequence for a single block.

I. Study Phase

In each study phase, all 16 words were presented visually in the middle of the screen for 3000-ms, followed by a fixation cross which appeared on the screen for 1000-ms. Participants were requested to remember as many words as they could.

II. Distraction task

During the delay between the study and free-recall phases, participants played a computerized version of the card game "Set". This game was chosen as a distraction task as it is engaging enough as to minimize rehearsal and is not verbal, therefore should not interfere with mnemonic processing of the word lists [4]. The length of the distraction task was one minute in the Short-Delay condition and twelve minutes in the Long-Delay condition. A Python implementation of the Set game was used, based on open-source code (www.github.com/aloverso/setgame). The goal of the game is to find as many sets as possible from a 3X3 matrix of 9 tiles. A valid set consists of three tiles that meet either of the

following criteria: (1) They all have the same shape, or they all have three different shapes; (2) They all have the same color, or they have three different colors; (3) They all have the same filling, or they have three different fillings. A scoring counter was placed on the screen. Scores were not analyzed as the task was intended solely for distraction and temporal manipulation.

III. Free recall test phase

Immediately following the distraction task instructions appeared on the screen requesting participants to type in words which they recalled from the most recent list, one word at a time in any order. Time for each item was unlimited, but the entire test session was limited to 30,000 ms.

IV. Justifications recall phase

Following the free-recall test phase, the recalled words were presented individually in the order in which they were recalled by the participant (both correct and incorrect recalls were presented). For each of the presented words participants were instructed to describe the information they used to recall that particular word. The screen for typing each Justification was displayed until participants finished typing and pressed 'Enter', or until the limit of 20,000 ms per word elapsed.

V. Post-Experiment Questionnaire.

Following completion of the experiment, participants were asked a few questions regarding the manner in which they performed the various stages of the experiment. Participants were asked if during the distraction task they rehearsed studied words or focused on the Set game.

Data Analysis

Seven participants gave justifications for fewer than half the items probed. These participants were excluded from all analyses concerning justifications. However, their data was included in all other analyses which did not take justifications into account, but only recall performance. Data were processed with in-house Matlab scripts [13] and statistical analyses were performed with R version 4.1.0 [14]. Generalized linear mixed model (GLMMs) & Linear mixed models (LMMs) were conducted with the lme4 package [15], and post-hoc analyses were conducted with the emmeans package [16].

Generalized Linear Mixed Models (GLMMs)

To overcome the greatly unbalanced numbers of items with vs. without justifications we conducted the analyses of recall metrics comparing items with and without justifications using mixed models. In line with recommendations for analyzing accuracy scores with a logistic mixed model [17], recall accuracy and pattern of errors were examined with a logistic mixed model. Serial Position, Temporal Factor scores and Semantic Factor scores were analyzed with linear mixed models.

Measures of Contextual Information

The use of the free recall paradigm enabled us to obtain several measures for item and contextual information. We briefly describe the relevant measures of contextual information. While no measure can be seen as purely representing one type or the other, certain measures are mainly reflective of contextual or item information.

Pattern of Errors. Errors in free-recall may be broadly divided into two categories: (1) Prior-list intrusions (PLIs) and (2) extra-list intrusions (ELIs). PLIs refer to words that were studied in earlier lists. Their recall is an error as participants are instructed to only recall words from

the most recent list. PLIs reflect cases in which individuals erred in reinstating the context of the most recent list, and instead recalled a word from a previous list.

Contrary to PLIs, ELIs refer to recall of words that did not appear during the experiment at all. ELIs are mostly semantically related to the previously-recalled item [18] and are associated with pre-existing lexical information regarding a list item. Importantly, in contrast to PLIs, ELIs are not accounted for in terms of contextual retrieval. Thus, it is only erroneous item-information that is involved in eliciting ELIs.

Serial Position. A recency effect, recalling items from the end of the list, is often observed in free recall [19,20]. This effect can be explained according to certain models in terms of contextual dynamics. The inner context at recall is most similar to the inner context during the study of recent items, as opposed to the context of less recent items. This similarity of context increases the likelihood for successfully retrieving recent items [21,22]. In our study we used a distraction task, to minimalize effects of Short-term memory, and focused on comparison of processes in Long-term memory, which may decrease or even eliminate the recency effect. However, we expected the probability of recalling recent items to be greater in the Short-Delay condition, where the context is more similar between their encoding and retrieval than in the Long-Delay condition.

Temporal Clustering. We used a widely accepted measure of temporal organization in recall - the Temporal Factor score [23]. This score was calculated for each participant in each delay condition. The Temporal Factor is a single score describing an individual's tendency to successively recall contiguous items. At each transition between two consecutively recalled words the absolute value of that transition's lag was calculated, as well as the absolute value for all possible lags. The actual lag was then given a Spearman's rank based on the possible lags (with lowest lag, i.e., immediately adjacent items, given the highest rank. Finally, the

transition's Temporal Factor score was calculated according to the equation: where R is the actual transition's rank, and N is the number of possible transitions. The scores were then averaged over all transitions yielding a single score for each participant in each delay condition. T-Factor scores range from 0 to 1 (highest reliance on contiguous temporal organization), with 0.5 indicating chance level, where half the transitions were temporally contiguous, and half were not.

This effect is explained in terms of temporal context—all information peripheral to the study-items that gradually changes over time (e.g., thoughts, association triggered by the study-items). The temporal contexts of two neighboring study-items are similar, and hence recall of one of these items is likely to trigger recall of its neighbor, which shares a relatively similar context.

Semantic Clustering. To explore semantic memory organization, we calculated individual Semantic Factor scores. A similar calculation was performed as that used for Temporal Factor scores. Here too each transition between two recalled words received a Semantic Factor score between 0 and 1 based on the following equation: in this case, N refers to the number of all possible transitions that could have been made between two words in the list, and R to the rank of the actual transition in terms of semantic distance between the two words recalled. Similarly to Temporal Factor scores, Semantic Factor scores have a value between 0 and 1, with a score of 0.5 signifying a chance level of semantic organization [23].

Semantic relatedness reflects previously acquired knowledge regarding the items themselves, rather than information regarding their one-shot episodic contexts. Thus, semantic organization is predominantly a measure of the degree to which item-related information drives recall.

Results

Free Recall

Recall Accuracy

The mean number of words correctly recalled in the Long-Delay condition (M_{Long} = 4.651, *SD* = 1.74,) was lower than in the Short-Delay condition (M_{Short} = 7.549, *SD* =1.58). This difference between conditions was evident in 100% of participants. A paired-samples t-test confirmed this difference to be statistically significant ($t(32) = 14.064$, $p < .001$, Cohen's d = 2.448), demonstrating an overall effect of temporal-delay on recall accuracy. This result replicates the findings of time-dependent forgetting in free recall [4], and displays an overall effect for time delay on memory retrieval, thus serving as a manipulation check for temporal delay.

Justifications

For each word recalled, participants described their "Justifications": subjective reflections regarding the information they used to recall that word. In addition to examining justifications' validity over time, we sought to establish that justifications are indeed reflective of retrieval of contextual information, as well as exploring how they interact with established effects in free recall.

Justifications and Accuracy

Justifications were initially coded into one of two categories: J+ (participants reported a justification regarding recall of that item) or J- (participants reported not having any such justification or giving no justification at all). A mean percentage of 85.3% (SD = 10.5%) of the items recalled included justifications.

To analyze recall-accuracy by justifications, we used a generalized linear mixed model for binomial outcomes ("GLMM" see Method), with the accuracy of recalls (correct/incorrect recall) as the dependent variable, delay condition (Short/Long) and justification category (J+/J-) as fixed independent variables, and participants as a random factor. Figure A2 shows the proportions of accurate recalls (out of all words recalled in each delay condition and justification category).

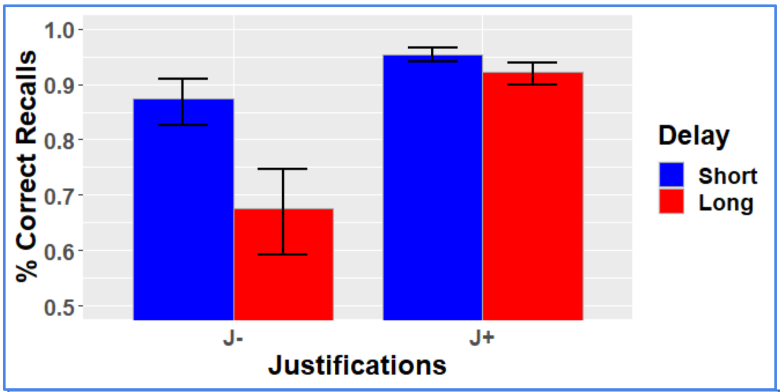

Figure A2. Percentage of correct recall by delay conditions and justifications. Error bars represent 95% confidence intervals around the

We initially included as a "base model" (M0) an empty within-person model accounting for participants' random intercepts (M0 fit: -2LL = 2414.2, df=2). A model with Delay (Short/Long) as a fixed effect, and participants' random intercepts ("M1"), yielded a significantly better model fit than M0 (M1 fit: -2LL = 2371.3, df=3; $\chi^2(1) = 342.904$, $p <$ .0001). In M1 the fixed effect of condition was significant (see Table 2) demonstrating an

overall effect of temporal-delay on recall accuracy, reaffirming the result described in the "Recall Accuracy" section.

A second model ("M2") including as fixed effects the main effect of Delay, main effect of Justifications, and the Delay*Justifications interaction, as well as participants' random intercepts, yielded a better model fit than M1, the model with only the main effect of Delay (M2 fit: 2254.8, df=5; $\chi^2(2) = 116.534$, $p < .0001$).

	M1			M2		
Predictors	*Odds Ratios*	*CI*	*p*	*Odds Ratios*	*CI*	*p*
Intercept	16.68	12.66 – 21.99	**<0.001**	6.90	4.75 – 10.02	**<0.001**
Delay [Long]	0.48	0.38 – 0.59	**<0.001**	0.30	0.20 – 0.45	**<0.001**
Justification [J+]				3.04	2.11 – 4.37	**<0.001**
Delay*Justification				1.86	1.14 – 3.05	**0.013**
**Random Effects**						
σ^2	3.29			3.29		
τ_{00}	0.32_{id}			0.29_{id}		
ICC	0.09			0.08		
N	26_{id}			26_{id}		
Observations	4245			4245		
Marginal R^2 / Conditional R^2	0.035 / 0.121			0.086 / 0.159		

Table A2. Accuracy by Delay (Short/Long) and justification categories (J-/J+). Predictor estimates in the Binomial GLMM are displayed here on the Odds-Ratio (OR) scale. The CI column displays 95% Confidence Intervals for the OR estimates.

In M2, there was a significant main effect for Delay, and a significant main effect for Justifications (see Table 2). The latter main effect indicates that accuracy was higher for words in which individuals reported an awareness of what triggered retrieval than for words lacking such an awareness. This result demonstrates that individuals' self-reported justifications are indicative of memory performance, thereby supporting the validity of justifications as a credible measure of mnemonic performance.

Finally, the Delay*Justifications interaction was also significant, demonstrating differing effects of delay on recall depending on the presence or lack of justifications (J+/J-).

Follow-up analyses showed significant differences between Short-Delay and Long-Delay conditions in both items with justifications (J+ : log odds-ratio = 0.579, odds-ratio = 1.78, z = 4.215, $p < .0001$) and without justifications (J- : log odds-ratio = 1.202, odds-ratio = 3.33, z = 5.713, $p < .0001$). However, an interaction contrast showed that the effect of delay was significantly higher in items without justifications, than those with justifications (log odds-ratio = 0.623, odds-ratio = 1.86, z = 2.487, $p = .013$). These results suggest that for items with justifications the degree of delay-dependent forgetting is lesser than for items without justifications. This demonstrates that for items recalled along with their accompanying justification (J+) the accuracy of recall remains relatively high after both short and long delays, whereas for items without justifications (J-) recall accuracy is considerably lowered after a long delay.

Justifications and Pattern of Errors

To examine the relation between justifications and measures reflecting context/item information, we considered two types of errors: Prior-list intrusions (PLIs) are words studied in previous lists. Extra-list intrusions (ELIs) are words not presented in any of the lists preceding the current recall test. PLIs are reflective of errors in context reinstatement, whereas ELIs reflect errors in retrieval of item-information (see Method). The proportions of error types (ELI/PLI) by delay and justifications categories are shown in Figure A3. We constructed a GLMM predicting whether a recall is an ELI or not by the fixed effects of Delay and Justifications (including participants' random intercepts). The first model ("ELI-1", see Table 3) included participants' random intercepts, and the fixed effect of Delay. This model yielded a significantly better model fit than an empty model including only participants' random intercepts (ELI-1 fit: -2LL = 2051.7, df=3; $\chi^2(1) = 24.8$, $p < .0001$). The second model ("ELI-2") Included the participants' random intercepts as well as the fixed

effects of Delay and Justifications, and the Delay*Justifications interaction. This model yielded a significantly better model fit than ELI-1 (ELI-2 fit: -2LL = 1966.7, df=5; $\chi^2(2) = 85.045$, $p < .0001$). In ELI-2, both fixed main effects of Delay, and Justifications were significant. However, the Delay*Justifications interaction was not significant. Follow-up analyses showed that there were significant differences between delay conditions both for items with justifications (J+ : log odds-ratio = -0.476, odds-ratio = 0.62, z = 3.159, p = .002), and for items without justifications (J- : log odds-ratio = -0.982, odds-ratio = 0.36, z = 4.343, $p < .0001$). On the descriptive level the difference by condition was greater for items without justifications than for items with justifications, however the difference did not reach significance (log odds-ratio = -0.506, odds-ratio = 0.603, z = 1.866, p = .062).

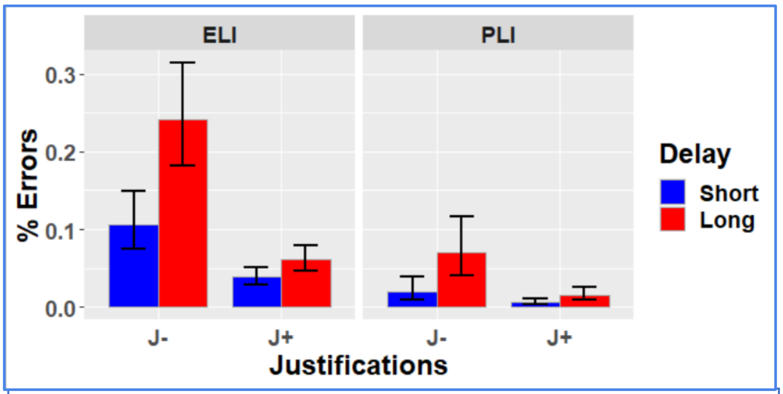

Figure A3. Percentage of Intrusions (ELIs / PLIs) by delay and justifications. Error bars represent 95% confidence intervals around the mean.

	ELI-1			ELI-2			PLI-1			PLI-2		
Predictors	*Odds Ratios*	*CI*	*p*	*Odds Ratios*	*CI*	*p*	*Odds Ratios*	*CI*	*p*	*Odds Ratios*	*CI*	*p*
Intercept	0.05	0.04 – 0.07	**<0.001**	0.12	0.08 – 0.18	**<0.001**	0.01	0.01 – 0.01	**<0.001**	0.02	0.01 – 0.04	**<0.001**
Delay [Long]	1.86	1.46 – 2.37	**<0.001**	2.67	1.71 – 4.16	**<0.001**	2.82	1.76 – 4.55	**<0.001**	3.76	1.71 – 8.29	**0.001**
Justification [J+]				0.34	0.23 – 0.50	**<0.001**				0.32	0.14 – 0.72	**0.006**
Delay*Justification				0.60	0.35 – 1.03	0.062				0.66	0.24 – 1.78	0.411
**Random Effects**												
σ^2	3.29			3.29			3.29			3.29		
τ_{00}	0.27_{id}			0.23_{id}			0.50_{id}			0.44_{id}		
ICC	0.08			0.07			0.13			0.12		
N	26_{id}			26_{id}			26_{id}			26_{id}		
Observations	4245			4245			4245			4245		
Marginal R^2 / Conditional R^2	0.025 / 0.099			0.072 / 0.134			0.064 / 0.187			0.103 / 0.208		

Table A3. Intrusions (ELIs/not PLIs/not accordingly) by Delay (Short/Long) and Justification categories (J-/J+).

We also constructed a GLMM for PLIs, predicting whether a recall is a PLI or not by delay and justification category and including participants’ random intercepts. The first

model (“PLI-1”) included participants’ random intercepts and the fixed effect of Delay. This model yielded a significantly better model fit than an empty model including only participants’ random intercepts (PLI-1 fit: -2LL = 692.40, df=3; $\chi^2(1) = 18.226$, $p < .0001$). The second model (“PLI-2”) included participants’ random intercepts as well as the fixed effects of Delay and Justifications, and the Delay*Justifications interaction. This model yielded a significantly better model fit than PLI-1 (PLI-2 fit: -2LL = 666.87, df=5; $\chi^2(2) = 25.532$, $p < .0001$). In PLI-2, both main effects of Delay, and Justifications were significant, but the Delay*Justifications interaction was not (see Table 3).

These results demonstrate a significant increase in the proportion of errors (ELIs and PLIs) in the Long-Delay condition compared to the Short-Delay condition. There are also more errors among the items without justifications than the items with justifications, in line with the results of justifications accuracy. These results help alleviate the concern that participants’ justifications may be confabulations, as the proportion of ELIs was lower in items with justifications than in items without justifications. Moreover, the increase in ELIs in the long delay condition was more pronounced (on the descriptive level) for items without justifications, than for items with justifications.

Justifications and Serial Position

To examine the occurrence of a recency effect by study condition and items with or without justifications we examined the serial position of items recalled. In line with previous

studies [24], the 16 item positions were split into 4 equal bins. Probability of recall by position bins is shown in Figure A4.

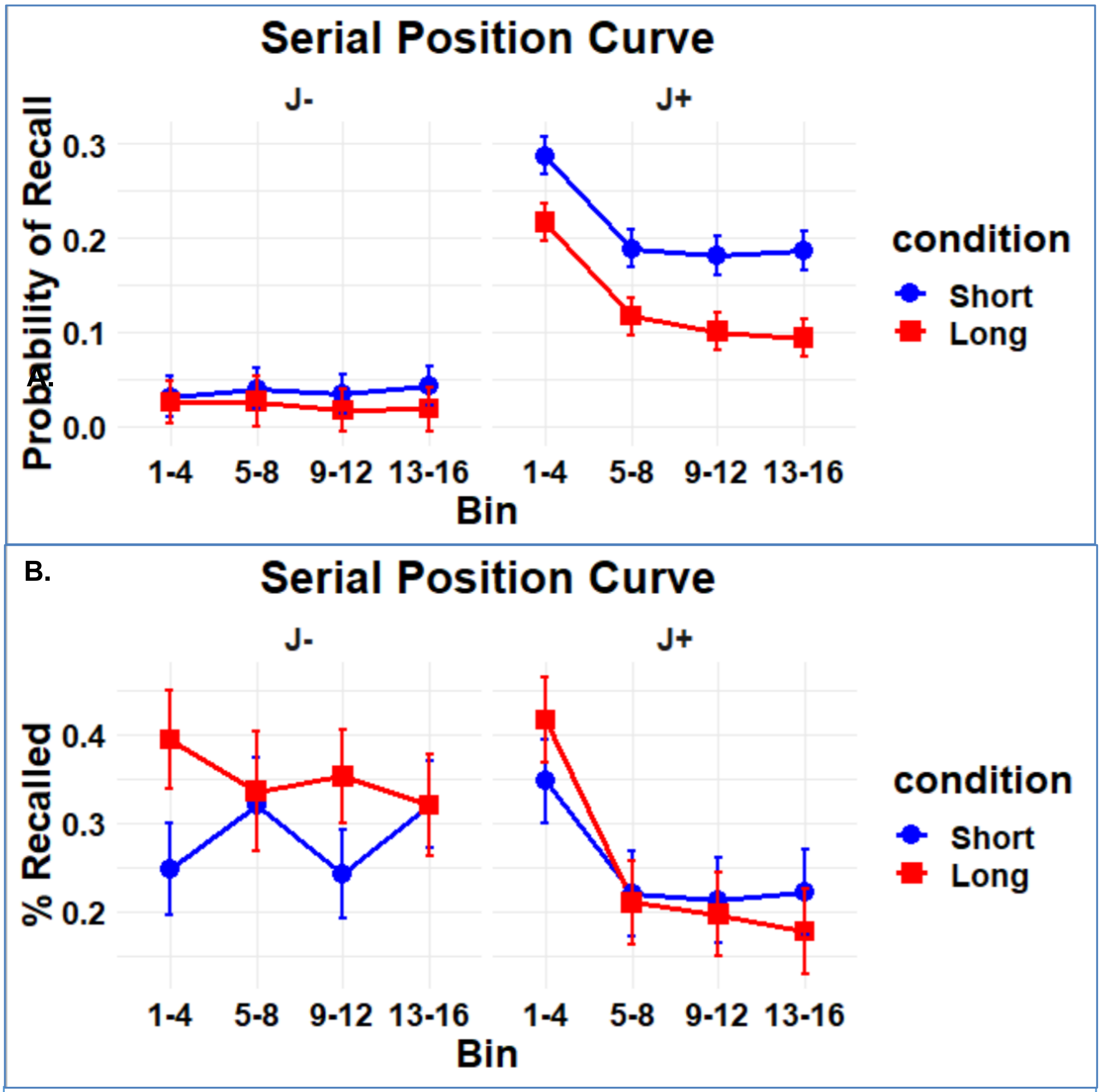

Figure A4. The Serial Position Curve split into four bins of serial position, for each delay condition and justification category. A. The probability of a word from a certain bin to be recalled. B. The proportion of words recalled from each bin in that delay condition and justification category. Error bars represent 95% confidence intervals around the mean.

A Linear Mixed Model ("LMM") was conducted, predicting the probability of recall, by the fixed effects of position (bins 1-4), delay, and justifications, as well as participants' random intercepts. The base model included participants' random intercepts and the fixed effect of Serial-Position. The first Model ("SPC-1") included participants' random intercepts and fixed effects of Serial-Position, as well as Justifications and the Serial-

Position*Justifications interaction. This model yielded a significantly better fit than the base model (SPC-1 fit: -2LL = -1074.03, df=10; $\chi^2(4) = 376.18$, $p < .0001$).

The second model ("SPC-2") added to SPC-1 the fixed effect of Delay as well as the Delay*Position and Delay*Justifications 2-way interactions and the 3-way Delay*Justifications*Position interaction. This model yielded a significantly better fit than SPC-1 (SPC-2 fit: -2LL = -1213.71, df=18; $\chi^2(8) = 139.69$, $p < .0001$). In SPC-2, the main effects of Serial-Position (F(3,330.94) = 31.778, p < .0001), Delay (F(1,331.19) = 95.856, p < .0001) and Justifications (F(1,331.84) = 863.616, p < .0001) were significant.

The Serial-Position*Justifications interaction was significant (F(3, 330.94) = 32.765, p < .0001). Recency effect, defined as the difference between the probability of recall in the 13-16 bin, and the 9-12 bin, was not significant in either items with or without justifications (J-: $t(332) = 0.508$, $p = .612$; J+: $t(330) = -0.090$, $p = .928$) demonstrating that the recency effect was greatly diminished by the distraction task. However, a post-hoc test of the probability of correct recalls revealed that the tendency to recall words from the 13-16 position bin (the last positions per list) was greater in the items with justifications than in the items without justifications ($t(331) = 11.572$, $p < .0001$). This result demonstrated a greater tendency to correctly recall end-of-list items in items accompanied by justifications than in items without justifications. Moreover, as Figure A4(B) shows, this is true also for the relative percentage of correct recalls for end-of-list items (out of correct recalls in that justifications category). Primacy effects, defined as the difference between probability of recall in the 1-4 bin and the 5-8 bin, were significant only for items with justifications, but not for items without justifications (J-: $t(332) = -0.440$, $p = .660$; J+: $t(330) = 11.137$, $p < .0001$), demonstrating that the simple Primacy effects were driven mainly by items with justifications.

The Justifications*Delay interaction was significant as well ($F(1,331.19) = 43.59$, $p < .0001$). Follow-up analyses show that the effect of Delay was considerably larger for the items with justifications than for items without justifications (J-: $t(332) = 2.104$, $p = .036$; J+: $t(330) = 12.553$, $p < .0001$).

In summary, the Serial-Position results indicate that the probability of recalling recent items was greater in the Short-Delay condition compared to the Long-Delay condition in line with forgetting over time of contextual information. In addition, the probability of recalling recent items was greater for items with justifications compared to items without justifications, and Primacy effects were found only in items with justifications. Thus, serial position effects, which are driven by the workings of context [21,22], are only found for items whose recall is accompanied by explicit retrieval of the internal context, as evidenced by the existence of justifications.

Justifications and Temporal Clustering

To examine the retrieval of temporal context and its relation to justifications, Temporal Factors scores were calculated in both study conditions, and in items with and without justifications. The Temporal Factor scores by Justifications and Delay conditions are presented in Figure A5 (right panel). An LMM was conducted, predicting the Temporal Factor score by the fixed effects of Delay and Justifications, as well as by participants' random intercepts. The base model included only participants' random intercepts. The first Model ("TF-1") included participants' random intercepts and the fixed effect of Justifications. This model yielded a significantly better fit than the base model (TF-1 fit: -2LL = -87.689, df=4; $\chi^2(1) = 7.56$, $p = .006$). The second model ("TF-2") included participants' random intercepts as well as the fixed effects of Delay and Justifications and the Delay*Justifications interaction. This model did not yield a significantly better model fit than TF-1 (TF-2 fit: -2LL = -87.867, df=6; $\chi^2(2) = 0.177$, $p = .914$). This result indicates that

Delay does not affect reliance on temporal order, replicating previous results [4,25]. In TF-1 the effect of Justifications was significant (t(782.48) = 2.753, p = .006), indicating the Temporal Factor score is significantly higher for Items with justifications than for items without justifications. This result, demonstrating that justifications are linked to the temporal context effect [23,26] also indicates that justifications are reflective of contextual information.

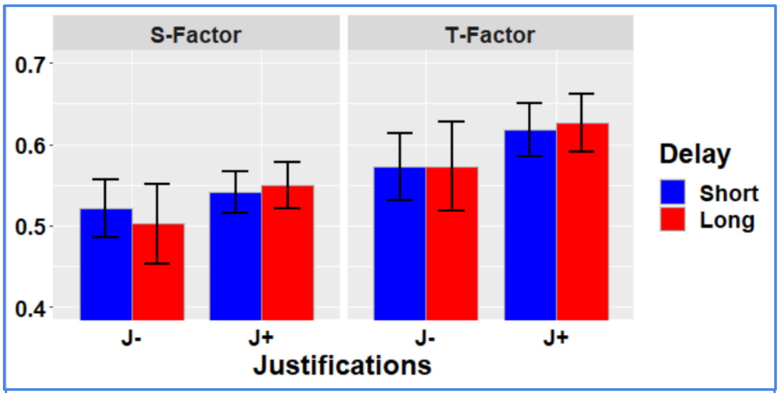

Figure A5. Semantic and Temporal Factor scores by delay conditions and justifications. Error bars represent 95% confidence intervals around the mean.

Justifications and Semantic Clustering

To examine the retrieval of semantic item-information and its relation to justifications, Semantic Factors scores were calculated in both study conditions, and in items with and without justifications. The Semantic Factor scores are presented in Figure A5 (left panel). An LMM was conducted predicting the Semantic Factor score by the fixed effects of Delay and Justifications, as well as participants' random intercepts. The base model included only participants' random intercepts. The first Model ("SF-1") included participants' random intercepts and fixed effects of Justifications. This model did not yield a significantly better fit than the base model (SF-1 fit: -2LL = -146.71, df=4; $\chi^2(1) = 2.919$, p = .088). The second model ("SF-2") included participants' random intercepts as well as the fixed effect of Delay. This model also did not yield a significantly better model fit than the base model (SF-2 fit: -2LL = -143.84, df=4; $\chi^2(1) = 0.05$, p = .828). This result indicates that Delay does not affect reliance on semantic associations, replicating previous findings [4]. As opposed to the Temporal Factor scores, results indicate there is no difference in the Semantic Factor scores between items with justifications and items without justifications (see Figure A5). This result

is in line with Semantic Factor scores reflecting item-information as opposed to contextual information [4], and again strengthens the notion that justifications are reflective of contextual-, but not item- information.

Justifications' Content

Justifications' Subjective Typology

The Justifications+ category was further broken down into different sub-categories according to mnemonic strategies (see Table 1 and Figure A6).

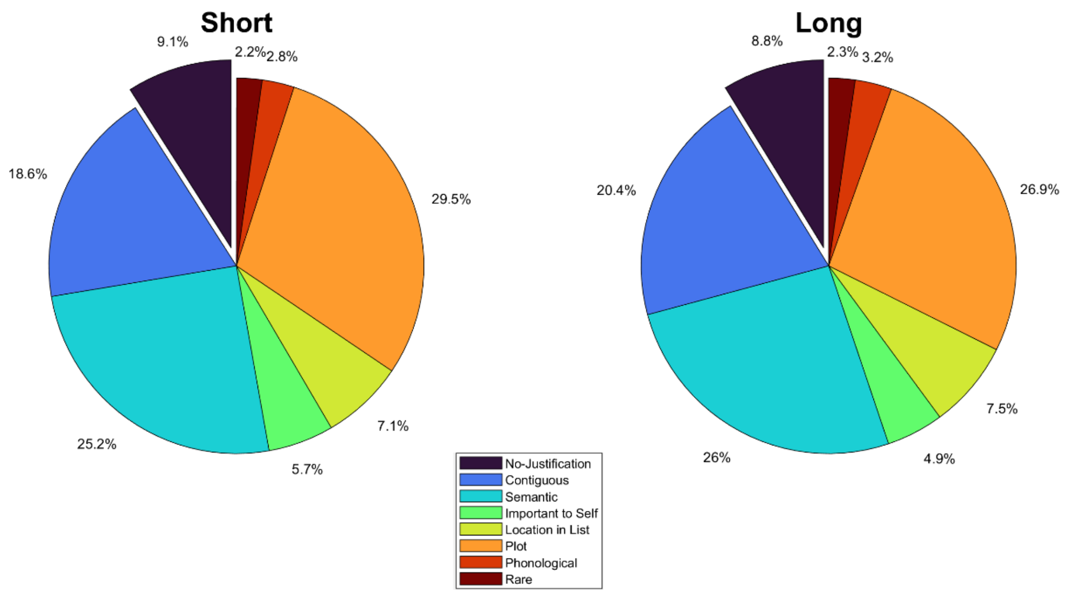

Figure A6. Proportions of justifications sub-categories in each delay condition.

The percentages of justifications in each category out of the sum of all justifications (per delay condition) were calculated. This was done to determine if there was a shift between the Short-Delay and Long-Delay conditions in the relative proportion of different types of justifications (e.g., different types of subjective information represented by justifications). The sums and percentages of each sub-category are described in Table 4. No such shift was observed, as the percentages of each justification type were virtually identical in the two delay conditions (see Figure A6). Bayesian paired sample t-tests were conducted, comparing the proportions of justifications in each category in the Short-Delay and Long-

Delay conditions. Bayes Factors (BFs) provided moderate support for the Null model for all but two categories, whose support was inconclusive (see Table 4).

		justification -	justification +						
		-	Contiguous	Semantic	Important to self	Location in list	Plot	Phonological	Rare
**Mean # Words**	Short	10.31	26.92	31.42	6.27	8.23	41.62	3.00	2.54
	Long	5.81	19.12	20.81	3.69	6	25.31	2.12	1.50
**Mean % words**	Short	0.09	0.19	0.25	0.06	0.07	0.29	0.03	0.02
	Long	0.09	0.20	0.26	0.05	0.08	0.27	0.03	0.02
**Short-Long BF_{01}**		4.683	1.775	4.358	3.328	4.266	2.203	4.320	4.790

Table A4. Sums and proportions of items in each justifications sub-category.

Thus, despite the effects of delay on recall accuracy (as shown above and as seen in the raw numbers of justifications per condition), the proportions of justifications in each category were not affected by delay. This shows that the prevalence of specific types of subjective information retrieved are not sensitive to passage of time, as the likelihood of each specific sub-category remains the same in both delays.

Justifications' Level of Detail

The number of details, word-related details, and number of abstract/concrete nouns were coded for each justification. Results are presented in Table 5 below. No differences were found in these measures between the Short-Delay and Long-Delay conditions. Bayesian analyses show moderate support in favor of the null hypothesis in all measures but the

number of concrete Details. Thus, the content of self-reports is also unaffected by delay. The level of detail, percentage of details related to the main event (i.e., the word recalled), and degree of abstract language are similar in both delay conditions, again demonstrating preservation of justification content over time delays.

	# DETAILS	# WORD-RELATED DETAILS	# ABSTRACT	# CONCRETE
**SHORT**	1.432	0.603	0.296	0.461
**LONG**	1.396	0.593	0.293	0.429
**Short-Long BF_{01}**	3.672	3.844	4.757	1.696

Table A5. Average justifications content by delay condition.

Justifications' Linguistic content

Words frequency scores per delay condition were calculated for all justifications (J+) and plotted using scattertext [12]. As seen in Figure A7, few words were distinctive to one delay condition or another. The frequency scores by delay conditions were very highly correlated (*pearson's* $r = 0.92$, $p < .001$), demonstrating that words frequency levels were similar across time delays.

Similarly, Cohen's d values for differences in word scores between conditions were low (all ds between -0.11 to 0.13), for low frequency as well as high frequency words (See Figure A8). These further strengthen the notion that the linguistic content of justifications remains highly similar across short and long time-delays.

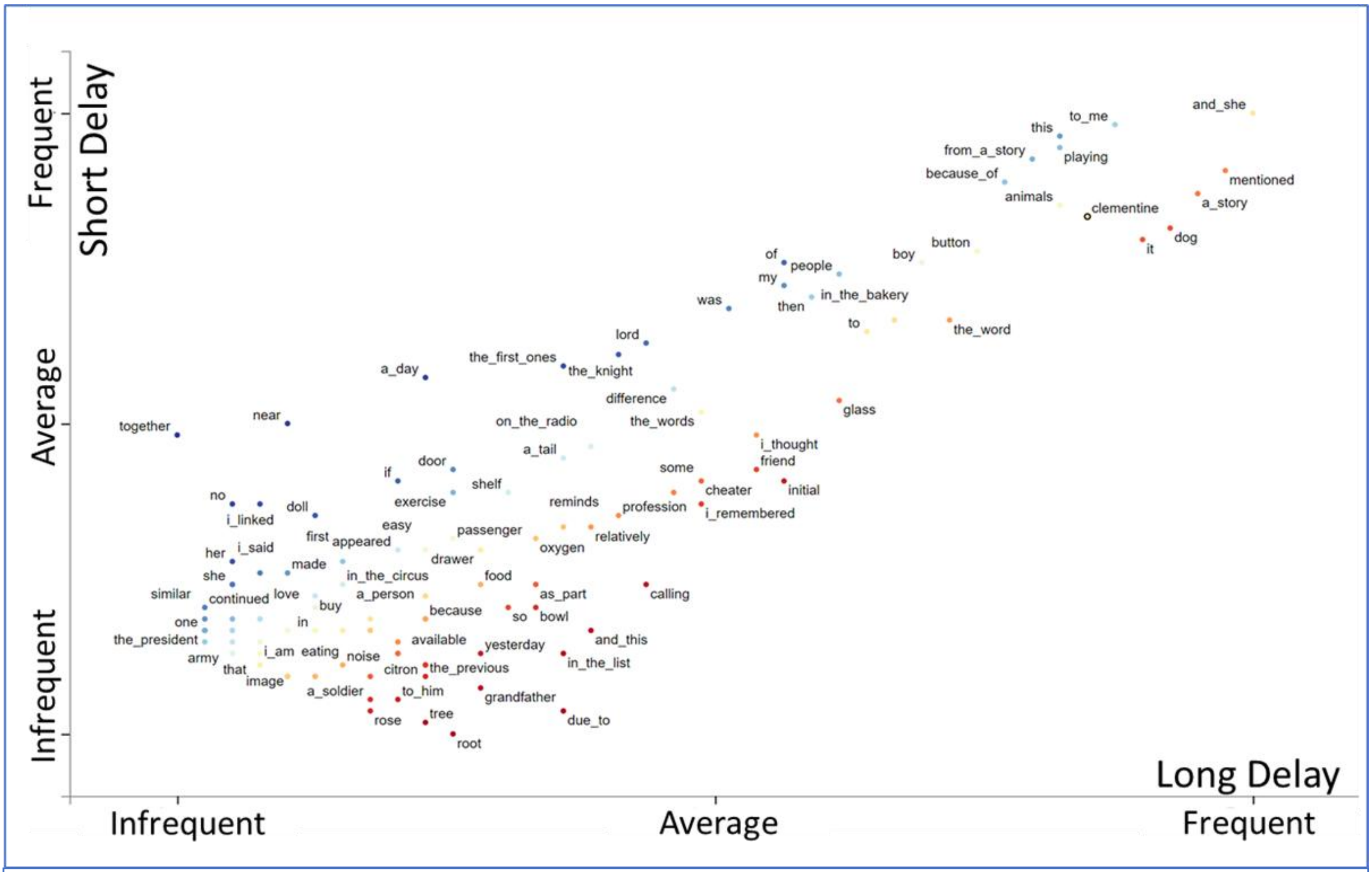

Figure A7. Justifications word frequencies by delay condition. Note: terms were scored as Hebrew unigrams and translated in the plot

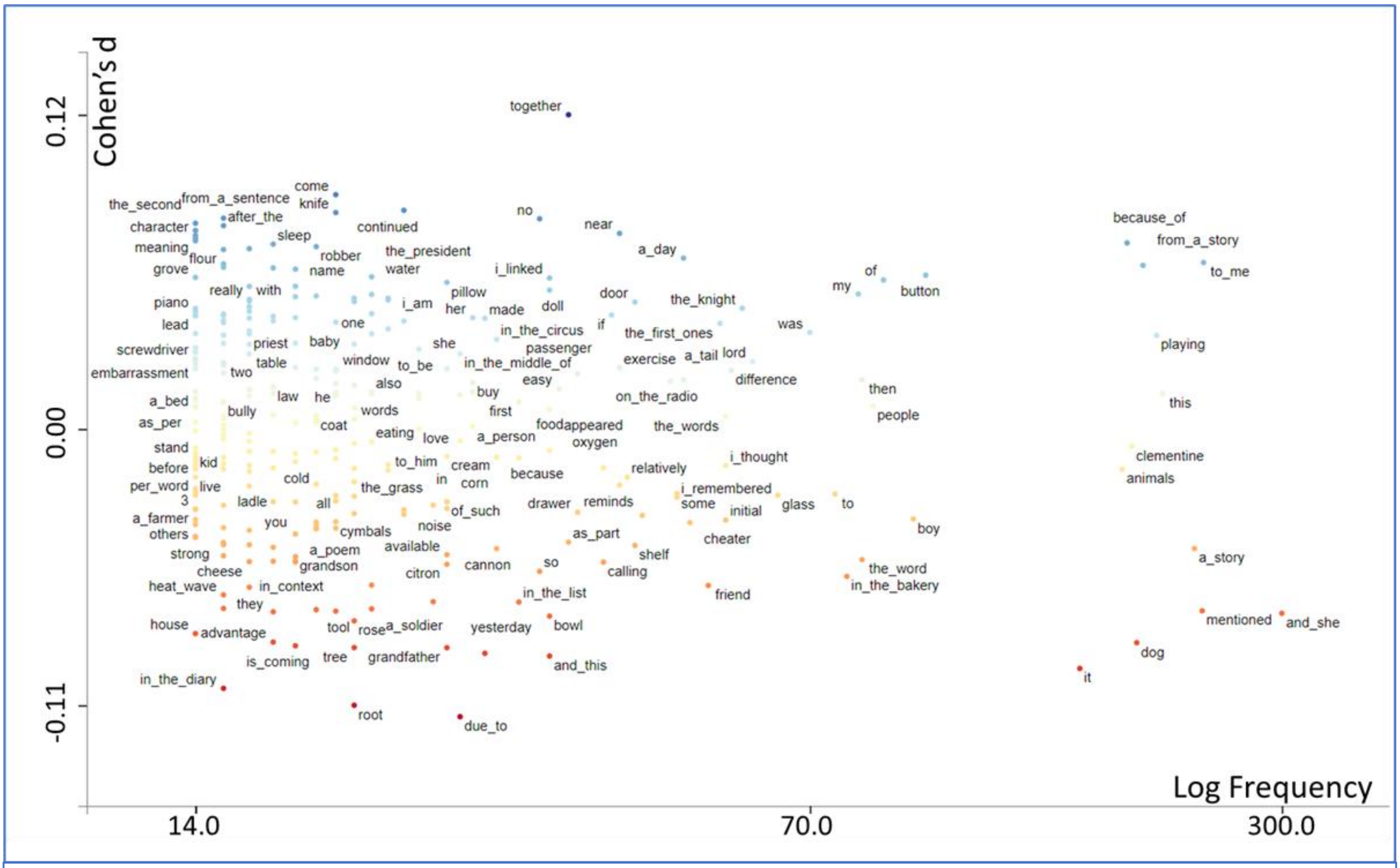

Figure A8. Effect sizes (Cohen's d) for differences in word frequency scores between delay conditions by word frequency.
Note: terms were scored as Hebrew unigrams and translated in the plot

Supplementary References

1. Rubenstein, O., Anaki, D., Henik, A., Drori, S. & Paran, Y. Hebrew free association norms. *Hebrew word norms [in Hebrew]* 17–34 (2005).
2. Kenett, Y. N., Kenett, D. Y., Ben-Jacob, E. & Faust, M. Global and local features of semantic networks: Evidence from the Hebrew mental lexicon. *PLoS One* (2011) doi:10.1371/journal.pone.0023912.
3. Kenett, Y. N., Levi, E., Anaki, D. & Faust, M. The semantic distance task: Quantifying semantic distance with semantic network path length. *J. Exp. Psychol. Learn. Mem. Cogn.* **43**, 1470–1489 (2017).
4. Gamoran, A., Greenwald-Levin, M., Siton, S., Halunga, D. & Sadeh, T. It's about time: Delay-dependent forgetting of item- and contextual-information. *Cognition* (2020) doi:10.1016/j.cognition.2020.104437.
5. Gilead, M., Liberman, N. & Maril, A. "I remember thinking...": Neural activity associated with subsequent memory for stimulus-evoked internal mentations. *Soc. Neurosci.* (2014) doi:10.1080/17470919.2014.902862.
6. Dobbins, I. G. & Kantner, J. The language of accurate recognition memory. *Cognition* (2019) doi:10.1016/j.cognition.2019.05.025.
7. Unsworth, N. Examining the dynamics of strategic search from long-term memory. *J. Mem. Lang.* (2017) doi:10.1016/j.jml.2016.09.005.
8. Levine, B., Svoboda, E., Hay, J. F., Winocur, G. & Moscovitch, M. Aging and autobiographical memory: Dissociating episodic from semantic retrieval. *Psychol. Aging* (2002) doi:10.1037/0882-7974.17.4.677.
9. Sekeres, M. J. *et al.* Recovering and preventing loss of detailed memory: Differential rates of forgetting for detail types in episodic memory. *Learning and Memory* (2016) doi:10.1101/lm.039057.115.

10. Robin, J. & Moscovitch, M. Details, gist and schema: hippocampal–neocortical interactions underlying recent and remote episodic and spatial memory. *Current Opinion in Behavioral Sciences* Preprint at https://doi.org/10.1016/j.cobeha.2017.07.016 (2017).
11. Winocur, G. & Moscovitch, M. Memory transformation and systems consolidation. *Journal of the International Neuropsychological Society* Preprint at https://doi.org/10.1017/S1355617711000683 (2011).
12. Kessler, J. S. Scattertext: a Browser-Based Tool for Visualizing how Corpora Differ. *arXiv [cs.CL]* (2017).
13. The Mathworks Inc. MATLAB - MathWorks. *www.mathworks.com/products/matlab* http://dx.doi.org/2016-11-26 (2016) doi:2016-11-26.
14. R Core Team. R: A language and environment for statistical computing. *R: A language and environment for statistical computing. R Foundation for Statistical Computing, Vienna, Austria* Preprint at (2020).
15. Bates, D., Mächler, M., Bolker, B. & Walker, S. Fitting Linear Mixed-Effects Models Using lme4. *Journal of Statistical Software* vol. 67 1–48 Preprint at https://doi.org/10.18637/jss.v067.i01 (2015).
16. Lenth, R., Singmann, H., Love, J., Buerkner, P. & Herve, M. *Estimated marginal means, aka least-squares means*. (2020).
17. Jaeger, T. F. Categorical data analysis: Away from ANOVAs (transformation or not) and towards logit mixed models. *J. Mem. Lang.* (2008) doi:10.1016/j.jml.2007.11.007.

18. Zaromb, F. M. *et al.* Temporal associations and prior-list intrusions in free recall. *Journal of Experimental Psychology: Learning Memory and Cognition* Preprint at https://doi.org/10.1037/0278-7393.32.4.792 (2006).

19. Bjork, R. A. & Whitten, W. B. Recency-sensitive retrieval processes in long-term free recall. *Cogn. Psychol.* (1974) doi:10.1016/0010-0285(74)90009-7.

20. Murdock, B. B. The serial position effect of free recall. *J. Exp. Psychol.* (1962) doi:10.1037/h0045106.

21. Sederberg, P. B., Howard, M. W. & Kahana, M. J. A Context-Based Theory of Recency and Contiguity in Free Recall. *Psychol. Rev.* (2008) doi:10.1037/a0013396.

22. Kahana, M. J., Sederberg, P. B. & Howard, M. W. Putting Short-Term Memory Into Context: Reply to Usher, Davelaar, Haarmann, and Goshen-Gottstein (2008). *Psychol. Rev.* (2008) doi:10.1037/a0013724.

23. Sederberg, P. B., Miller, J. F., Howard, M. W. & Kahana, M. J. The temporal contiguity effect predicts episodic memory performance. *Memory and Cognition* (2010) doi:10.3758/MC.38.6.689.

24. Brooks, B. M. Primacy and recency in primed free association and associative cued recall. *Psychon. Bull. Rev.* (1999) doi:10.3758/BF03210838.

25. Berens, S. C., Richards, B. A. & Horner, A. J. Dissociating memory accessibility and precision in forgetting. *Nature Human Behaviour* (2020) doi:10.1038/s41562-020-0888-8.

26. Howard, M. W. & Kahana, M. J. A distributed representation of temporal context. *J. Math. Psychol.* (2002) doi:10.1006/jmps.2001.1388.

Supplementary Note 2 | Pilot study 2 + 3

Pilot Study 2: 1-day Lag

Method

Participants

The study included 120 online participants via Prolific, 13 were excluded due to failing to return for the second part of the study. Participants were compensated with a monetary award of 0.75£ for part 1 and 0.8 £ for part 2 (according to a rate of 7.5/ 9.6 £ per hour accordingly). All participants were English speakers from childhood.

Materials

Lists

The experiment consisted of two lists of 16 words each. Words are 3-10 letters long nouns and adjectives, selected from the Penn Electrophysiology of Encoding and Retrieval Study word pool (PEERS [1,2]) , which contains 1638 words (available at: http://memory.psych.upenn.edu/files/wordpools/PEERS_wordpool.zip). Five lists have been constructed such that temporal and semantic contributions to recall can be dissociated. In each list, varying degrees of semantic relatedness occur at both adjacent and distant serial positions. Semantic relatedness was determined using the word association space ("WAS" 47). The WAS similarity values were used to group words into four similarity bins (high: $\cos\theta > 0.7$; medium-high: $0.4 < \cos\theta < 0.7$; medium-low: $0.14 < \cos\theta < 0.4$; low: $\cos\theta < 0.14$). For each list, 8 pairs of words with high semantic similarity were selected. Next, words within the lists were organized such that members of a pair did not appear adjacent to one another. In addition, no adjacent pair had a high similarity. This list construction method was used in previous studies [3]. For each list, four different ordering schemes of the words within the list

were created. The lists and ordering schemes were counterbalanced across participants, with random assignment to one of the lists and schemes.

Justifications

Participants' self-reports of their recall process, or "justifications", were collected similarly to Pilot study 1 (See Appendix A). In this study they were reported in English. Following each recall test phase –in the separate justifications condition– or following each word recalled –in the mixed condition– participants were asked to describe the information they used to recall the word. Before the justification phase (or before recall in the interleaved condition), participants were given the instruction: "You will now be shown again the words you just remembered. (/Following each word recalled you will be shown that word again). Please explain in as much detail as possible why you think this particular word appeared in the study phase. Consider any factors that you think might justify your recollection of the word". During the justification / recall phase each individual justification was preceded with the instruction: "Please describe in as much detail as possible why you believe the word above appeared in the study phase". Justifications were recalled and typed by participants who were cued with each word recalled during the word-recall phase.

Justifications' detail

For a fine-grained analysis of the effects of time on justifications, each justification was scored using the automated Autobiographical Interview [4–6], in which a given text is classified as internal or external to the event described, providing a score of the proportion of internal/external detail.

Justifications' linguistic content

The number of words per justification were counted. In addition, justifications' level of concreteness were scored using a concreteness norms dictionary [7], providing an average concreteness score per justification. Levels of language abstraction may be reflective of transformation from a contextually-detailed memory to a verbatim gist memory [8,9]. In addition, dictionaries of certitude and tentativeness from the Linguistic Inquiry and Word Count tool (LIWC-22 [10]) were used to measure degree of confidence/certainty expressed in justifications.

Experimental procedure

Participants recruited from Prolific were randomly assigned to one of two delay-order conditions. All participants studied and were tested on two word lists: one with a short delay of 1.5 minutes and one with a long delay of 1 day between study and test. In one condition the short delay preceded the long delay and the other vice versa. Participants were also randomly assigned to one of two Recall-Order conditions: either providing justifications one at a time following each word recalled as part of the recall test phase (mixed condition), or providing justifications for all items recalled in a separate justifications phase (separate condition).

The experiment consisted of a total of two blocks. Each block included 4 stages: I. study phase, II. Time Interval / Distraction task, III. Free-recall, IV. Self-report of justifications. In the mixed Recall-Order condition phases III and IV were interleaved in the same phase.

I. Study Phase

In each study phase, each of the 16 words was presented in the middle of the screen for 4000-ms, followed by a fixation cross which appeared on the screen for 1000-ms. Participants were instructed to remember as many words as possible.

II. Distraction task

In the 1.5 minutes delay conditions, during the delay between the study and free-recall phases, participants solved math problems of the form X+Y+Z = ? (where X,Y,Z are single digit integers) between study and test. This was chosen as a distraction task as it is engaging enough as to minimize rehearsal and is not verbal, therefore should not interfere with mnemonic processing of the word lists [25].

In the 1-day delay conditions, after the study phase, participants received instructions to revisit the experiment website after 1 day for a test phase and received a reminder by email.

The order of the delay conditions (Short/Long) was counterbalanced across participants. Participants who started with the Short-Delay condition, performed the Short-Delay study, distraction task, and test, and then immediately the Long-Delay study on the first day. They then returned after a delay of 1 day to perform the Long-Delay test. Participants who started with the Long-Delay condition, performed the Long-Delay study on the first day and returned after a delay of 1 day to perform the Long-Delay test, and then immediately performed the Short-Delay study, distraction task, and Short-Delay test.

III. Free recall test phase

Immediately following the distraction task or upon revisiting the website after the time interval, participants were requested to type in words which they recalled from the study phase, one word at a time in any order. Time for recall was unlimited, and the recall phase continues until participants press a button indicating they do not recall any more words.

IV. Justifications recall phase

Following the free-recall test phase, the recalled words were presented individually in the order in which they were recalled by the participant (both correct and incorrect recalls were presented). For each of the presented words, participants were instructed to describe why they believe that word appeared (see "Justifications" above). Time for reporting justifications was unlimited. Following each individual justification participants were requested to indicate their level of confidence in their correct recall on a scale from 1 to 6. This too was untimed. In the mixed Recall-Order condition, each word in the recall phase was followed by providing a justification and a confidence score for that word (instead of a separate justification phase).

Results

Free Recall

Duration of Study-Test delay

The mean number of words correctly recalled in the Long-Delay condition (M_{Long} = 2.73, *SD* = 3.41) was lower than in the Short-Delay condition (M_{Short} = 6.63, *SD* = 4.17). This difference between delays was evident in 79.4% (85 out of 107) of participants. A paired-samples t-test confirmed this difference to be statistically significant ($t(106) = 9.70$, $p < .001$, Cohen's d = 0.94, $BF_{10} = 2.28 \times 10^{13}$), demonstrating an overall effect of temporal-delay on recall accuracy. This result replicates the findings of time-dependent forgetting in free recall [3], and displays an overall effect for time delay on memory retrieval, thus serving as a manipulation check for temporal delay.

Delay Order

To examine effects of delay order (i.e., short-delay first or last) on recall accuracy, we conducted a Bayesian 2X2 mixed ANOVA for number of correct recalls by Delay-Duration (Short / Long) and Delay-Order (Short-Long / Long-Short). The analysis demonstrated that the best fit for the data was a model including both main effects of Delay-Duration and Delay-Order and their interaction. See Table 1.

Table 1. Bayesian ANOVA Model Comparison for Correct Recalls by Delay-Duration and Delay-Order

Model	BF10	Log BF10
Delay-Order + Subject	0.30	-1.21
Delay-Duration + Subject	5.08×10^{13}	31.56
Delay-Order + Delay-Duration + Subject	1.89×10^{13}	30.57
Delay-Order X Delay-Duration + Subject	7.41×10^{15}	37.16

The data demonstrated very strong evidence in favor of the main effect of Delay-Duration ($BF_{inclusion} = 5.36 \times 10^{13}$), weak evidence against the main effect of Delay-Order ($BF_{inclusion} = 0.37$), and very strong evidence in favor of the Delay-Duration X Delay-Order interaction ($BF_{inclusion} = 393.15$; See Figure A1).

Follow up Bayesian t-tests demonstrated within the Short-Long Delay-Order condition very strong evidence for the difference between number of correct recalls by Delay-Duration ($BF_{10} = 1.85 \times 10^{13}$), and within the Long-Short Delay-Order condition strong evidence for the difference between number of correct recalls by Delay-Duration ($BF_{10} = 84.61$), with the calculated Bayes Factors differing by several orders of magnitude, demonstrating a considerably more pronounced effect for Delay-Duration within the Short-Long Delay-Order condition.

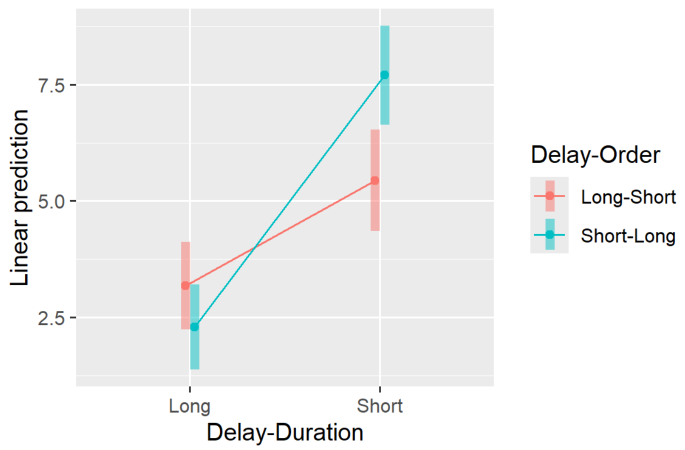

Figure A1. Number of Correct Recalls by Delay-Duration and Delay-Order.

Recall Order

To examine effects of retrieval order (i.e., justifications after each word recalled or in a separate phase) we conducted a Bayesian 2X2 mixed ANOVA for number of correct recalls by Delay-Duration (Short / Long), and Recall-Order (justification elicitation Mixed / Separated from recall). The analysis demonstrated that the best fit for the data included the main effect Delay-Duration only. See Table 2.

Table 2. Bayesian ANOVA Model Comparison for Correct Recalls by Delay-Duration and Recall-Order

Model	BF10	Log BF10
Recall-Order + Subject	0.50	-0.70
Delay-Duration + Subject	5.08×10^{13}	31.56
Recall-Order + Delay-Duration + Subject	3.23×10^{13}	31.11
Recall-Order X Delay-Duration + Subject	2.48×10^{13}	30.84

The data demonstrated very strong evidence in favor of the main effect of Delay-Duration ($BF_{inclusion} = 5.55 \times 10^{13}$), weak evidence against the main effect of Recall-Order ($BF_{inclusion} = 0.64$), and weak evidence against the Delay-Duration X Recall-Order interaction ($BF_{inclusion} = 0.77$).

Taken together these findings suggest a main effect for Delay-Duration on number of correct recalls, which is considerably more pronounced in the Delay-Order which performed first the Short Delay followed by the Long Delay. Recall-Order did not meaningfully affect recall.

Temporal Clustering

Temporal Factor scores the tendency to rely on temporal contiguity for recollection. Temporal Factor scores were significantly above chance levels for both the short delay (M = 0.65, SD = 0.17, $t(49) = 6.10$, $p < .001$, Cohen's d = 1.74, $BF_{10} = 8.96 \times 10^4$) and the long delay ($M$ = 0.63, SD = 0.21, $t(49) = 4.63$, $p < .001$, Cohen's d = 1.32, $BF_{10} = 765.61$). No significant differences were found between delay conditions ($t(49) = 0.51$, $p = .613$, Cohen's d = 0.07), a Bayesian paired t-test demonstrated positive evidence against differences between conditions ($BF_{01} = 5.75$).

A 2X2 Bayesian mixed ANOVA including Delay-Duration and Delay-Order, demonstrated that the best fit was for the null model, without main effects of Delay-Duration, Delay-Order or their interactions. See Table 3.

Table 3. Bayesian ANOVA Model Comparison for Temporal Factor Scores by Delay-Duration and Delay-Order

Model	BF10	Log BF10
Delay-Order + Subject	0.56	-0.58
Delay-Duration + Subject	0.24	-1.44
Delay-Order + Delay-Duration + Subject	0.13	-2.03
Delay-Order X Delay-Duration + Subject	0.03	-3.36

A 2X2 Bayesian mixed ANOVA including Delay-Duration and Recall-Order, demonstrated that the best fit was for the null model, without main effects of Delay-Duration, Recall-Order or their interactions. See Table 4.

Table 4. Bayesian ANOVA Model Comparison for Temporal Factor Scores by Delay-Duration and Recall-Order

Model	BF10	Log BF10
Recall-Order + Subject	0.30	-1.20
Delay-Duration + Subject	0.24	-1.44
Recall-Order + Delay-Duration + Subject	0.07	-2.64
Recall-Order X Delay-Duration + Subject	0.03	-3.61

These findings suggest Temporal Factor Scores were above chance levels and did not differ by Delay-Duration, Delay-Order or Recall-Order.

Justifications

For each word recalled, participants described their memory justifications, relating contextual information which may have been instrumental in driving recollection.

Number of Words

The number of words included in justifications was submitted to a 2X2 Bayesian mixed ANOVA including Delay-Duration and Delay-Order. The best fit was demonstrated for the model including only the main effect of Delay-Duration. See Table 5.

Table 5. Bayesian ANOVA Model Comparison for Word Count by Delay-Duration and Delay-Order

Model	BF10	Log BF10
Delay-Order + Subject	0.31	-1.17
Delay-Duration + Subject	9809.53	9.19
Delay-Order + Delay-Duration + Subject	3408.12	8.13

Delay-Order X Delay-Duration + Subject	883.63	6.78

The data demonstrated very strong evidence in favor of the main effect of Delay-Duration ($BF_{inclusion}$ = 1.01 X 10^4), weak evidence against the main effect of Delay-Order ($BF_{inclusion}$ = 0.35), and positive evidence against the Delay-Duration X Delay-Order interaction ($BF_{inclusion}$ = 0.26). Follow-up Bayesian paired t-tests demonstrated very strong evidence (BF_{10} = 1.08 X 10^4) for justifications following a Short delay (M = 6.30, SD = 3.97) being shorter than justifications following a Long delay (M = 8.97, SD = 6.10).

A 2X2 Bayesian mixed ANOVA including Delay-Duration and Recall-Order, demonstrated the best fit was for the model including only both main effects of Delay-Duration and Recall-Order, but not their interaction. See Table 6.

Table 6. Bayesian ANOVA Model Comparison for Word Count by Delay-Duration and Recall-Order

Model	BF10	Log BF10
Recall-Order + Subject	58.48	4.07
Delay-Duration + Subject	9809.53	9.19
Recall-Order + Delay-Duration + Subject	5.71 X 10^5	13.25
Recall-Order X Delay-Duration + Subject	2.03 X 10^5	12.22

The data demonstrated very strong evidence in favor of the main effect of Delay-Duration ($BF_{inclusion}$ = 9.76 X 10^3), strong evidence in favor of the main effect of Recall-Order ($BF_{inclusion}$ = 58.20), and weak evidence against the Delay-Duration X Recall-Order interaction ($BF_{inclusion}$ = 0.36). Follow-up Bayesian paired t-tests demonstrated very strong evidence (BF_{10} = 804.95) for justifications in the Mixed Recall-order condition (M = 5.85,

SD = 3.65) being shorter than justifications in the Separate Recall-order condition (M = 9.25, SD = 6.03).

These findings suggest Justification lengths (by word count) were impacted by Delay Duration and Recall Order, additively and without an interaction effect. Delay order did not affect Justification length.

Justifications' detail

The level of detail in the stories was analyzed using the automated scorer based on the autobiographical interview method [6]. This model counts the degree of internal and external details per text. Internal details are episodic details pertaining to the main event of the narrative. External details are mostly non-episodic (though they may also contain episodic details not pertaining to the main event [6]). The score of internal details is composed of the proportion of internal information identified by the scoring tool. The proportions of internal details of justifications were submitted to a 2X2 Bayesian mixed ANOVA, including Delay-Duration and Delay-Order. The best fit was demonstrated for the model including the main effect of Delay-Duration. See Table 7.

Table 7. Bayesian ANOVA Model Comparison for Internal Details by Delay-Duration and Delay-Order

Model	BF10	Log BF10
Delay-Order + Subject	0.23	-1.45
Delay-Duration + Subject	1.35	0.30
Delay-Order + Delay-Duration + Subject	0.32	-1.14
Delay-Order X Delay-Duration + Subject	0.12	-2.15

The data demonstrated weak evidence in favor of the main effect of Delay-Duration ($BF_{inclusion}$ = 1.35), positive evidence against the main effect of Delay-Order ($BF_{inclusion}$ =

0.24), and weak evidence against the Delay-Duration X Delay-Order interaction ($BF_{inclusion}$ = 0.36).

A 2X2 Bayesian mixed ANOVA including Delay-Duration and Recall-Order, demonstrated the best fit was for the model including the main effect of Delay-Duration. See Table 8.

Table 8. Bayesian ANOVA Model Comparison for Internal Details by Delay-Duration and Recall-Order

Model	BF10	Log BF10
Recall-Order + Subject	0.47	-0.75
Delay-Duration + Subject	1.35	0.30
Recall-Order + Delay-Duration + Subject	0.64	-0.44
Recall-Order X Delay-Duration + Subject	0.15	-1.88

The data demonstrated weak evidence in favor of the main effect of Delay-Duration ($BF_{inclusion}$ = 1.35), weak evidence against the main effect of Recall-Order ($BF_{inclusion}$ = 0.47), and positive evidence against the Delay-Duration X Recall-Order interaction ($BF_{inclusion}$ = 0.24).

These findings suggest Justification's level of detail was unaffected by main effects of Delay-Order and Recall-Order, and only anecdotally affected by the main effect of Delay-Duration.

Concreteness

Justifications' levels of concreteness were submitted to a 2X2 Bayesian mixed ANOVA including Delay-Duration and Delay-Order. The best fit was demonstrated for the model including only the main effect of Delay-Duration. See Table 9.

Table 9. Bayesian ANOVA Model Comparison for Concreteness by Delay-Duration and Delay-Order

Model	BF10	Log BF10
Delay-Order + Subject	0.23	-1.48
Delay-Duration + Subject	2.85	1.05
Delay-Order + Delay-Duration + Subject	0.66	-0.42
Delay-Order X Delay-Duration + Subject	0.14	-1.94

The data demonstrated weak evidence in favor of the main effect of Delay-Duration ($BF_{inclusion}$ = 2.85), positive evidence against the main effect of Delay-Order ($BF_{inclusion}$ = 0.23), and positive evidence against the Delay-Duration X Delay-Order interaction ($BF_{inclusion}$ = 0.22).

A 2X2 Bayesian mixed ANOVA including Delay-Duration and Recall-Order, demonstrated the best fit was for the model including only the main effect of Delay-Duration. See Table 10.

Table 10. Bayesian ANOVA Model Comparison for Concreteness by Delay-Duration and Recall-Order

Model	BF10	Log BF10
Recall-Order + Subject	0.26	-1.35
Delay-Duration + Subject	2.85	1.05
Recall-Order + Delay-Duration + Subject	0.75	-0.29
Recall-Order X Delay-Duration + Subject	0.18	-1.70

The data demonstrated weak evidence in favor of the main effect of Delay-Duration ($BF_{inclusion}$ = 2.85), positive evidence against the main effect of Recall-Order ($BF_{inclusion}$ = 0.26), and positive evidence against the Delay-Duration X Recall-Order interaction ($BF_{inclusion}$ = 0.24).

These findings suggest anecdotal effects for Delay-Duration on justification concreteness levels and no effect for, Delay-Order or Recall-Order.

Certitude / Tentativeness

The average degree of Certitude and Tentativeness in words used in justifications was calculated using dictionaries of these attributes from LIWC 2022 [10].

A 2X2 Bayesian mixed ANOVA including Delay-Duration and Delay-Order, demonstrated that the best fit was for the null model, without main effects of Delay-Duration, Delay-Order or their interactions. See Table 11.

Table 11. Bayesian ANOVA Model Comparison for Certitude by Delay-Duration and Delay-Order

Model	BF10	Log BF10
Delay-Order + Subject	0.66	-0.41
Delay-Duration + Subject	0.22	-1.50
Delay-Order + Delay-Duration + Subject	0.15	-1.92
Delay-Order X Delay-Duration + Subject	0.04	-3.20

A 2X2 Bayesian mixed ANOVA including Delay-Duration and Recall-Order, demonstrated that the best fit was for the null model, without main effects of Delay-Duration, Recall-Order or their interactions. See Table 12.

Table 12. Bayesian ANOVA Model Comparison for Certitude by Delay-Duration and Recall-Order

Model	BF10	Log BF10
Recall-Order + Subject	0.59	-0.53
Delay-Duration + Subject	0.22	-1.50
Recall-Order + Delay-Duration + Subject	0.13	-2.04
Recall-Order X Delay-Duration + Subject	0.03	-3.59

Justifications' levels of Tentativeness were submitted to a 2X2 Bayesian mixed ANOVA including Delay-Duration and Delay-Order. The best fit was demonstrated for the null model without main effects of Delay-Duration, Delay-Order or their interactions. See Table 13.

Table 13. Bayesian ANOVA Model Comparison for Tentativeness by Delay-Duration and Delay-Order

Model	BF10	Log BF10
Delay-Order + Subject	0.32	-1.14
Delay-Duration + Subject	0.29	-1.25
Delay-Order + Delay-Duration + Subject	0.09	-2.39
Delay-Order X Delay-Duration + Subject	0.02	-3.85

A 2X2 Bayesian mixed ANOVA including Delay-Duration and Recall-Order, demonstrated the best evidence for the null model, without main effects of Delay-Duration, Recall-Order or their interactions. See Table 14.

Table 14. Bayesian ANOVA Model Comparison for Tentativeness by Delay-Duration and Recall-Order

Model	BF10	Log BF10
Recall-Order + Subject	0.55	-0.53
Delay-Duration + Subject	0.29	-1.25
Recall-Order + Delay-Duration + Subject	0.17	-1.78
Recall-Order X Delay-Duration + Subject	0.06	-2.87

These findings suggest Justifications certitude and tentativeness were unaffected by Delay-Duration, Delay-Order or Recall-Order.

Unigram frequencies

The contents of justifications were further analyzed by counting the frequency of unigrams (single words) within justifications in each condition. The frequency of each unigram was determined by a simple count over all justifications per condition. The frequency of unigrams was submitted to a 2X2 Bayesian mixed ANOVA, including Delay-Duration and Delay-Order. The best fit was demonstrated for the model including both main effects of Delay-Duration and Delay-Order and the Delay-Duration X Delay-Order interaction. See Table 15.

Table 15. Bayesian ANOVA Model Comparison for Unigram Frequencies by Delay-Duration and Delay-Order

Model	BF10	Log BF10
Delay-Order + Word	0.23	-1.46
Delay-Duration + Word	1.71×10^6	14.36
Delay-Order + Delay-Duration + Word	4.35×10^5	12.98
Delay-Order X Delay-Duration + Word	1.08×10^7	16.20

The data demonstrated very strong evidence in favor of the main effect of Delay-Duration ($BF_{inclusion}$ = 1.75 X 10^6), positive evidence against the main effect of Delay-Order ($BF_{inclusion}$ = 0.25), and strong evidence in favor of the Delay-Duration X Delay-Order interaction ($BF_{inclusion}$ = 24.91). Follow up Bayesian t-tests demonstrated within the Short-Long Delay-Order condition very strong evidence for the difference between number of correct recalls by Delay-Duration (BF_{10} = 1.36 X 10^3), and within the Long-Short Delay-Order condition positive evidence for the difference between number of correct recalls by Delay-Duration (BF_{10} = 12.74), with the calculated Bayes Factors differing by a multiple of ~100, demonstrating a considerably more pronounced effect for Delay-Duration within the Short-Long Delay-Order condition.

A 2X2 Bayesian mixed ANOVA including Delay-Duration and Recall-Order, demonstrated the best evidence for the model including both main effects of Delay-Duration and Recall-Order but without their interaction. See Table 16.

Table 16. Bayesian ANOVA Model Comparison for Unigram Frequencies by Delay-Duration and Recall-Order

Model	BF10	Log BF10
Recall-Order + Word	2.21×10^{9}	21.52
Delay-Duration + Word	170.85	5.14
Recall-Order + Delay-Duration + Word	2.35×10^{12}	28.49
Recall-Order X Delay-Duration + Word	2.17×10^{12}	28.41

The data demonstrated very strong evidence in favor of the main effect of Delay-Duration ($BF_{inclusion} = 1.07 \times 10^{3}$), very strong evidence in favor of the main effect of Recall-Order ($BF_{inclusion} = 1.37 \times 10^{10}$), and weak evidence against of the Delay-Duration X Recall-Order interaction ($BF_{inclusion} = 0.92$).

Taken together these analyses suggest a main effect for Delay-Duration on justification content in the form of unigram frequencies, which is considerably more pronounced in the Delay-Order which performed first the Short Delay followed by the Long Delay. Recall-Order also demonstrated a main effect on justification content, but without interaction with Delay-Duration.

Numeric Confidence

Following the recall of justifications, participants were instructed to designate their confidence in their correct recall of the item on a scale from 1 to 6.

Confidence by study conditions

The numeric confidence scores were submitted to a 2X2 Bayesian mixed ANOVA, including Delay-Duration and Delay-Order. The best fit was demonstrated for the model including only the main effect of Delay-Duration. See Table 17.

Table 17. Bayesian ANOVA Model Comparison for Confidence Scores by Delay-Duration and Delay-Order

Model	BF10	Log BF10
Delay-Order + Subject	0.29	-1.24
Delay-Duration + Subject	8.38×10^6	15.94
Delay-Order + Delay-Duration + Subject	2.82×10^6	14.85
Delay-Order X Delay-Duration + Subject	8.15×10^5	13.61

The data demonstrated very strong evidence in favor of the main effect of Delay-Duration ($BF_{inclusion} = 8.67 \times 10^6$), weak evidence against the main effect of Delay-Order ($BF_{inclusion} = 0.34$), and positive evidence against the Delay-Duration X Delay-Order interaction ($BF_{inclusion} = 0.29$). Follow-up Bayesian paired t-tests demonstrated very strong evidence ($BF_{10} = 7.10 \times 10^6$) for greater numeric confidence following a Short delay (M = 5.54, SD = 0.81) than following a Long delay (M = 4.62, SD = 1.42).

A 2X2 Bayesian mixed ANOVA, including Delay-Duration and Recall-Order demonstrated the best fit for the model including only the main effect of Delay-Duration. See Table 18.

Table 18. Bayesian ANOVA Model Comparison for Confidence Scores by Delay-Duration and Recall-Order

Model	BF10	Log BF10
Recall-Order + Subject	0.30	-1.20
Delay-Duration + Subject	8.38×10^6	15.94

Recall-Order + Delay-Duration + Subject	$2.90 X 10^6$	14.88
Recall-Order X Delay-Duration + Subject	$9.23 X 10^5$	13.74

The data demonstrated very strong evidence in favor of the main effect of Delay-Duration ($BF_{inclusion}$ = $8.38 X 10^6$), weak evidence against the main effect of Recall-Order ($BF_{inclusion}$ = 0.35), and positive evidence against the Delay-Duration X Recall-Order interaction ($BF_{inclusion}$ = 0.32).

These findings suggest Numeric confidence scores were considerably decreased by greater Delay-Duration, but not affected by Delay-Order or Recall-Order.

Math Problems Task

The short delay consisted of 90 seconds of answering addition problems consisting of 3 integers between 1-9. The number of correctly answered problems ranged between 0-43 (Median = 17, Mean = 17.6, SD = 8.5). The number of correctly answered problems was not significantly correlated with the number of correct recalls following a short delay ($r(104) = 0.12$, $p = .217$), correct recalls following a long delay ($r(104) = -0.03$, $p = .740$), the temporal factor scores in the short delay ($r(47) = 0.05$, $p = .853$) or the temporal factor scores in the long delay ($r(47) = -0.03$, $p = .752$).

Pilot Study 3: 1-Week Lag

Method

Participants

The study included 119 online participants via Prolific, 32 were excluded due to failing to return for the second part of the study. Participants were compensated with a

monetary award of 0.9£ for part 1 and 1.05£ for part 2 (according to a rate of 7.7/9£ per hour accordingly). All participants were English speakers from childhood.

Study design was similar to study 2 detailed above, with the exception of the time delay for the long condition, which consisted of a 1-week delay as opposed to 1-day.

Results

Free Recall

Duration of Study-Test delay

The mean number of words correctly recalled in the Long-Delay condition (M_{Long} = 1.19, *SD* = 2.61) was lower than in the Short-Delay condition (M_{Short} = 5.98, *SD* = 3.72). A paired-samples t-test confirmed this difference to be statistically significant ($t(79) = 10.08$, $p < .001$, Cohen's d = 1.13), demonstrating an overall effect of temporal-delay on recall accuracy. This result replicates the findings of time-dependent forgetting in free recall [3], and displays an overall effect for time delay on memory retrieval, thus serving as a manipulation check for temporal delay.

Due to the low number of items recalled following a long delay of 1 week (A Mean of 1.19 correct recalls), the lag of 1 week was deemed too long for the sake of the current paradigm.

Supplementary References

1. Lohnas, L. J. & Kahana, M. J. Parametric effects of word frequency in memory for mixed frequency lists. *J. Exp. Psychol. Learn. Mem. Cogn.* **39**, 1943–1946 (2013).
2. Healey, M. K. & Kahana, M. J. Is memory search governed by universal principles or idiosyncratic strategies? *J. Exp. Psychol. Gen.* **143**, 575–596 (2014).
3. Gamoran, A., Greenwald-Levin, M., Siton, S., Halunga, D. & Sadeh, T. It's about time: Delay-dependent forgetting of item- and contextual-information. *Cognition* **205**, 104437 (2020).
4. Levine, B., Svoboda, E., Hay, J. F., Winocur, G. & Moscovitch, M. Aging and autobiographical memory: dissociating episodic from semantic retrieval. *Psychol. Aging* **17**, 677–689 (2002).
5. Sekeres, M. J. *et al.* Recovering and preventing loss of detailed memory: differential rates of forgetting for detail types in episodic memory. *Learn. Mem.* **23**, 72–82 (2016).
6. van Genugten, R. D. I. & Schacter, D. L. Automated scoring of the autobiographical interview with natural language processing. *Behav. Res. Methods* **56**, 2243–2259 (2024).
7. Brysbaert, M., Warriner, A. B. & Kuperman, V. Concreteness ratings for 40 thousand generally known English word lemmas. *Behav. Res. Methods* **46**, 904–911 (2014).
8. Robin, J. & Moscovitch, M. Details, gist and schema: hippocampal–neocortical interactions underlying recent and remote episodic and spatial memory. *Current Opinion in Behavioral Sciences* **17**, 114–123 (2017).
9. Winocur, G. & Moscovitch, M. Memory transformation and systems consolidation. *J. Int. Neuropsychol. Soc.* **17**, 766–780 (2011).
10. Boyd, R. L., Ashokkumar, A., Seraj, S. & Pennebaker, J. W. The development and psychometric properties of LIWC-22. University of Texas at Austin. Preprint at (2022).
