# Supplementary material for: Memory justifications provide valid indicators of retrieval accuracy across time
Source: Commun Psychol. 2025 Dec 31;4:10. doi: 10.1038/s44271-025-00378-4 (PMC12820377; doi:10.1038/s44271-025-00378-4)
Supplement: Supplementary file 3 — Reporting Summary [file 44271_2025_378_MOESM3_ESM.pdf]

nature portfolio

Corresponding author(s): DBPR COMMSPSYCHOL-23-0046C

Last updated by author(s): Nov 14, 2025

Reporting Summary

Nature Portfolio wishes to improve the reproducibility of the work that we publish. This form provides structure for consistency and transparency in reporting. For further information on Nature Portfolio policies, see our Editorial Policies and the Editorial Policy Checklist.

Statistics

For all statistical analyses, confirm that the following items are present in the figure legend, table legend, main text, or Methods section.

n/a	Confirmed	
☐	☒	The exact sample size (*n*) for each experimental group/condition, given as a discrete number and unit of measurement
☐	☒	A statement on whether measurements were taken from distinct samples or whether the same sample was measured repeatedly
☐	☒	The statistical test(s) used AND whether they are one- or two-sided<br>*Only common tests should be described solely by name; describe more complex techniques in the Methods section.*
☐	☒	A description of all covariates tested
☐	☒	A description of any assumptions or corrections, such as tests of normality and adjustment for multiple comparisons
☐	☒	A full description of the statistical parameters including central tendency (e.g. means) or other basic estimates (e.g. regression coefficient) AND variation (e.g. standard deviation) or associated estimates of uncertainty (e.g. confidence intervals)
☐	☒	For null hypothesis testing, the test statistic (e.g. *F*, *t*, *r*) with confidence intervals, effect sizes, degrees of freedom and *P* value noted<br>*Give P values as exact values whenever suitable.*
☐	☒	For Bayesian analysis, information on the choice of priors and Markov chain Monte Carlo settings
☐	☒	For hierarchical and complex designs, identification of the appropriate level for tests and full reporting of outcomes
☐	☒	Estimates of effect sizes (e.g. Cohen's *d*, Pearson's *r*), indicating how they were calculated

Our web collection on statistics for biologists contains articles on many of the points above.

Software and code

Policy information about availability of computer code

Data collection	data collection was performed on a custom private website.
Data analysis	Data analysis was performed using R version 4.2.1

For manuscripts utilizing custom algorithms or software that are central to the research but not yet described in published literature, software must be made available to editors and reviewers. We strongly encourage code deposition in a community repository (e.g. GitHub). See the Nature Portfolio guidelines for submitting code & software for further information.

Data

Policy information about availability of data

All manuscripts must include a data availability statement. This statement should provide the following information, where applicable:

- Accession codes, unique identifiers, or web links for publicly available datasets
- A description of any restrictions on data availability
- For clinical datasets or third party data, please ensure that the statement adheres to our policy

The pilot study data is freely available at the Open Science Framework (OSF) website (https://osf.io/3wu5a/). All study materials (e.g., word lists) and all behavioral data collected via the online study will be anonymized and made freely available at the same location.

Research involving human participants, their data, or biological material

Policy information about studies with human participants or human data. See also policy information about sex, gender (identity/presentation), and sexual orientation and race, ethnicity and racism.

Reporting on sex and gender	Sex was not considered in this study. Gender was self reported by participants in pilot studies and is reported in the sample description. Gender was not considered in the study analysis. The analyses and findings in the study are not impacted by sex or gender in any consequential way. The findings in the study are relevant across sex and gender identity.
Reporting on race, ethnicity, or other socially relevant groupings	No information on race ethnicity or social groups was gathered or reported in the study as it has no bearing on study analyses or results. The study findings are relevant across social groups
Population characteristics	No population characteristics were included as covariates in the study
Recruitment	Pilot study 1: Participants were university students who participated for course credit or monetary compensation<br>Pilot study 2+3: Participants were online workers recruited from the Prolific platform who participated for monetary compensation.
Ethics oversight	The proposed research complies with ethical regulations and was approved by the institutional review board at <DBPR> University. The participants will provide written consent prior to the research. Participants will be provided with monetary compensation according to the rate of £8 per hour.

Note that full information on the approval of the study protocol must also be provided in the manuscript.

Field-specific reporting

Please select the one below that is the best fit for your research. If you are not sure, read the appropriate sections before making your selection.

☐ Life sciences ☒ Behavioural & social sciences ☐ Ecological, evolutionary & environmental sciences

For a reference copy of the document with all sections, see nature.com/documents/nr-reporting-summary-flat.pdf

Behavioural & social sciences study design

All studies must disclose on these points even when the disclosure is negative.

Study description	A quantitative study on behavioral results of human memory after short and long delays, and computational linguistic analysis of participants self reported memory justifications.
Research sample	University students in pilot study 1, Prolific online workers in pilot studies 2 and 3 and in proposed main study
Sampling strategy	Random sampling from prolific online platform
Data collection	Data are collected from online participants who respond by keyboard on a laptop computer.
Timing	pilot study 1: November 2018 to April 2019.<br>Pilot study 2+3 July-August 2023
Data exclusions	Pilot study 1: No data were excluded<br>Pilot study 2: 13 participants (of 120) were excluded due to failing to return for the second part of the study.<br>Pilot study 3: 32 participants (of 119) were excluded due to failing to return for the second part of the study.
Non-participation	No participants dropped out of the study.
Randomization	Pilot study 1: participants were not assigned to groups, analyses were all within-participants<br>Pilot study 2+3: Participants were randomly assigned to counterbalanced study groups

Reporting for specific materials, systems and methods

We require information from authors about some types of materials, experimental systems and methods used in many studies. Here, indicate whether each material, system or method listed is relevant to your study. If you are not sure if a list item applies to your research, read the appropriate section before selecting a response.

Materials & experimental systems

n/a	Involved in the study
☒	☐ Antibodies
☒	☐ Eukaryotic cell lines
☒	☐ Palaeontology and archaeology
☒	☐ Animals and other organisms
☒	☐ Clinical data
☒	☐ Dual use research of concern
☒	☐ Plants

Methods

n/a	Involved in the study
☒	☐ ChIP-seq
☒	☐ Flow cytometry
☒	☐ MRI-based neuroimaging

Plants

Seed stocks	N/A
Novel plant genotypes	N/A
Authentication	N/A
